# Supplementary material for: In Search of Covariates of HIV-1 Subtype B Spread in the United States—A Cautionary Tale of Large-Scale Bayesian Phylogeography
Source: Viruses. 2020 Feb 5;12(2):182. doi: 10.3390/v12020182 (PMC7077180; doi:10.3390/v12020182)
Supplement: Supplementary file 1 [file viruses-12-00182-s001.pdf]

Supplementary Materials: In Search for Covariates of HIV-1 Subtype B Spread in the United States – A Cautionary Tale of Large-scale Bayesian Phylogeography.

Samuel L. Hong, Simon Dellicour, Bram Vrancken, Marc A. Suchard, Michael T. Pyne, David R. Hillyard, Philippe Lemey, Guy Baele

1. Description of study population

Our full dataset contains a heterogeneous spatial and temporal distribution of sequences. This is to be expected from habitual sampling bias and the underlying differences in population sizes between US states. This bias is most clear when looking at the number of sequences collected throughout the years, where we have less than 1,000 sequences per year prior to 2007, and a multi-fold increase in the years after (Figure S1). The distribution of sequences by location is also highly heterogeneous throughout time, with 99% of the sequences coming from NY during 2004 to 2006.

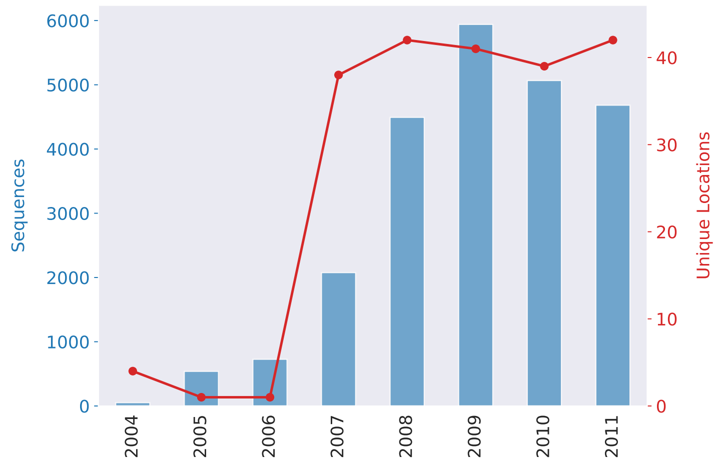

Figure S1. Number of sequences and unique sampling locations by year. The number of sequences collected each year (blue) increases dramatically after 2007. This coincides with an increase in the number of locations (red) being sampled in this data set.

To examine the differences in sampling density across the country (Figure S2B), we took the number of sequences collected in each state (Figure S2A), and divided it by the average number of individuals living with HIV between 2004–2011 [1]. Doing so shows considerable variability in the rate of sampling, with the number of samples per 100 HIV positive individuals per state ranging from 0–19.41. Regardless of sampling density, when looking at the spatial distribution of collected sequences within the entire time period, we see that the distribution of sequences by collection location roughly follows that of individuals living with HIV in each state (Figures S2 C and D). We can also use these values to identify locations that are over and under represented in the dataset. We do so by looking at the difference between the proportion of sequences collected from each location (Figure S2D), and the proportion of HIV positive individuals living in each state (Figure S2C), and see a mean difference of only 0.0004, and a standard deviation of 0.02. From this, we can identify four over-represented states (Georgia, Indiana, Massachusetts, Texas), — defined as having a difference in proportions above one standard deviation — and five under-represented states (California, Florida, Maryland, New York, and Pennsylvania). Regardless, when comparing the spatial distribution of sequences after our two-step subsampling procedure, we see that it closely matches that of the HIV population.

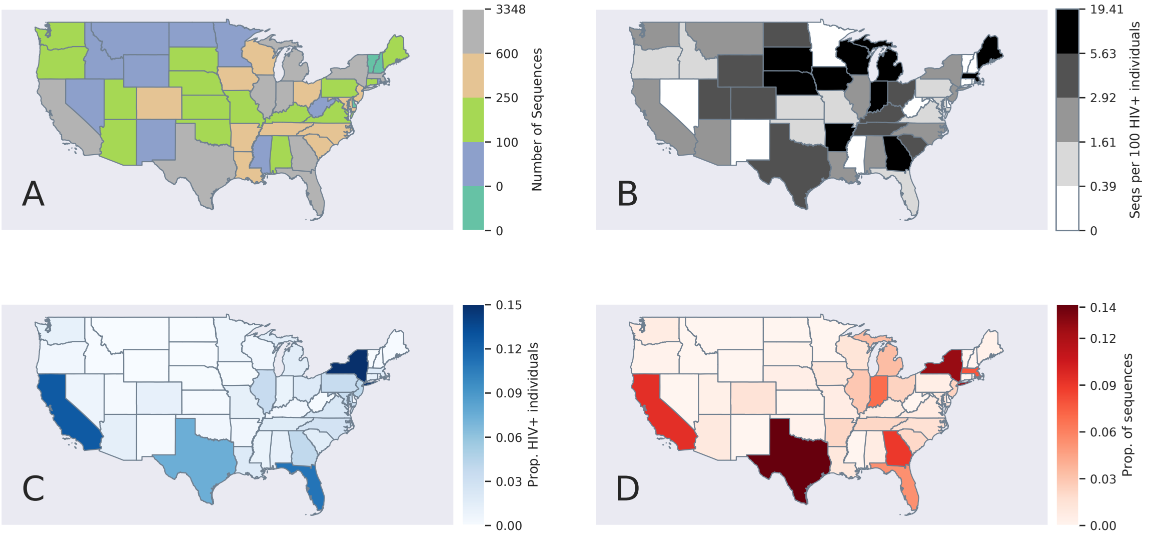

Figure S2. (A) Number of sequences collected from each state. No sequences were collected from the states of Alaska, Delaware, Hawaii, New Hampshire and Vermont. (B) Number of sequences collected from each state per 100 individuals living with HIV. (C) Proportion of the total number individuals living with HIV in the United States during 2004-2011 by location. (D) Proportion of the total number of sequences collected by location.

2. Predictors of HIV spread across state borders

We considered a total of 38 potential predictors in each phylogeographic reconstruction. An origin and destination predictor was specified for covariates that were state-specific, rather than pairwise. Pseudocounts were added to predictors with zero values, to ensure strictly positive values before log-transformation and standardization to a mean of 0 and a variance of 1. The potential predictors of HIV spatial spread we considered for the GLM diffusion model can be grouped into the following categories:

- **Mobility**: Mobility predictors are pairwise measures that describe the flow of people into and out of each state. The predictors we included in this category are aviation flux and commuting flow.

  We define aviation flux as the average number of passengers per day on flights between each pair of locations. We constructed an aviation flux matrix by first downloading all the air traffic data

between 2004 and 2011 from the Bureau of Transportation Statistics website [2], and aggregating the number of passengers in flights between states in the continental US.

Commuting flow refers to the number of individuals that live in one state but work in a different one. We constructed this matrix by downloading data on the county-level commuting flows from 2006-2010 from the United States Census Bureau's American Community Survey (ACS) [3] and restricting our matrix to commuting distances of less than 150 miles. This was done to ensure that the commuting matrix did not include potential flight commuting. Pairwise distances between counties were downloaded for the year of 2010 from the National Bureau of Economic Research [4], and used to aggregate the county flows into between-state movements.

- **Distances**: Distance predictors are pairwise measures that quantify the geographic distance between two locations. The potential distance predictors we included in the model are adjacency, simulated distance, and environmental distances.

  Adjacency between locations was incorporated as a binary matrix that represents states sharing a border with a value of 1 and 0 otherwise. This predictor was not log-transformed or standardized. We constructed this matrix from a shape file downloaded from the US Census Bureau [5] by looking at polygons with overlapping points.

  The simulated distance predictor is a matrix of average geodesic distances between two discrete locations. This distance measure was chosen as a more realistic alternative over centroid great-circle distances. The simulated distance matrix was constructed by drawing a number of points from each state corresponding to the number of sequences in each dataset. The locations of each point followed a mean population density raster [6] such that points were more likely to be drawn from areas with higher density. The distance between two states was then defined to be the average between all pairs of points between two states across 100 replicates.

  Environmental distances represent the distance between two points by taking into account inaccessible regions using a circuit theory framework [7]. In this framework, pairwise connectivity can be approximated by estimating pairwise electric resistance measures between locations. They incorporate environmental rasters as grids of electric resistance or conductance to study the connectivity among locations. This allows us to have a more realistic representation of the path needed to traverse when moving from one point to another. We compute these distances using the Circuitscape software [8,9]. We introduced two predictors to account for environmental distances in this project: an inaccessibility distance, created using an inaccessibility raster [10], and a null Circuitscape distance that we use as a negative control [11]. These predictors were considered only for the datasets of up to 1,000 taxa, since calculating these distances was too computationally intensive for larger sample sizes.
- **Economic**: We tested the role of economic data as a potential driver of spatial spread by including measures of gross domestic product (GDP) per capita, income inequality, and poverty as predictors. These measures were state-specific, so we specified both an origin and destination predictor for each of them.

  The GDP per capita predictor was constructed as an average from GDP per capita numbers from 2004-2011 obtained from the US Bureau of Economic Analysis [12].

  The poverty predictor was the multi-year average proportion of people in the Poverty Universe during the 2005-2011 time period. The Poverty Universe is defined as all persons determined to be in poverty status by the Census Bureau, except unrelated individuals under the age of 15. The proportions were made from a table with the multi-year averages of individuals living in poverty and the total number of people in each state, downloaded using the Current Population Survey (CPS) Table Creator tool from the US Census Bureau website [13].

  We incorporated the Gini index of income inequality into our model as a measure of inequality within each state. The Gini index is a statistical measure of income dispersion used to represent

the distribution of income within a place. A Gini index of 0 represents a perfectly equal population where everyone receives the same income, while a Gini index of 1 corresponds to a perfectly unequal society in which a single individual receives 100% of the total income, and the remaining people receive none. The average Gini index from 2006-2011 was obtained from the ACS [14], and used as a predictor.

- **Demographic**: We tested four demographic measures as potential predictors of spatial spread: census population, proportion of black population, proportion of men who have sex with men (MSM), and rates of incarceration. African Americans and gay men are disproportionately affected by HIV compared to other groups [15], and HIV prevalence in correctional populations is approximately five times that of the general adult population [16], which drove us to include these potential predictors into our model.

  Multi-year averages for total population and black population were obtained using the CPS Table Creator tool for the 2004-2011 time period [13]. The percentage of MSM in each state during 2009-2013 was obtained from [17].

  We included average rates of incarceration during the 2004-2011 time period for each state. We downloaded the prisoners bulletin reports for each year from the US Department of Justice's Bureau of Justice Statistics to obtain the number of prisoners in each state [18]. The average rate per 1,000 individuals was then computed using the CPS values for average state population size.

- **Education**: Education-related state-specific predictors were included to see if they play a role in the spread of HIV. Measures of educational attainment and sexual education were considered, since HIV is a sexually transmitted disease.

  We measured educational attainment by looking at the average proportion of people with high school or equivalent levels of education during the period of 2004 to 2011, with data from CPS [13].

  For sexual education measures, we included the percentage of secondary schools in which teachers taught the following topics in a required course in any of the grades 9-12 during the 2009-2010 school year: i) how to correctly use a condom, and ii) how HIV and other sexually transmitted diseases are transmitted. To this end, we used the data from the US Center for Disease Control's School Health Profiles 2010 survey [19].

  Percentages for the states of Colorado, Illinois, and New Mexico were missing in the 2010 dataset, and were imputed by using a regression between the 2010 values and the mean percentages using the 2012, 2014, and 2016 surveys.

- **Health**: We included the following state-specific measures of health as potential predictors in the model: proportion of uninsured people, Hepatitis C virus (HCV) prevalence, mortality rates from drug overdoses, and presence of syringe exchange programs. We included these predictors since HIV and HCV share a route of transmission, and injection drug use increases the risk for HIV infection.

  The average proportion of uninsured individuals in each state was obtained from ACS data for the years of 2004-2011 [20].

  Hepatitis C virus prevalence estimates for the year 2010 were included using the values from [21].

  We created a dichotomous variable to describe the presence or absence of syringe exchange programs, with 2012 data from amfAR [22].

  We followed [23] to obtain the drug overdose mortality rates for cocaine and heroin from the US Center for Disease Control [24].

- **Sampling Bias**: To account for the effect of sampling bias in the geographic history reconstruction, we included an additional origin and destination predictor based on the residuals for the regression of number of sequences, against the average number of individuals living with HIV in each state. For the datasets larger than 1,000 taxa, we also included the number of sequences as a predictor to absorb potential spurious inclusion of the predictors from the increased sampling bias. Our aim is not to demonstrate a role for sample sizes in phylogeography, but by explicitly including them as predictive variables, we raise the credibility that other predictors are not included in the model because of sampling bias.

3. Connectivity of phylogeographic reconstructions on the PDA subsampled datasets

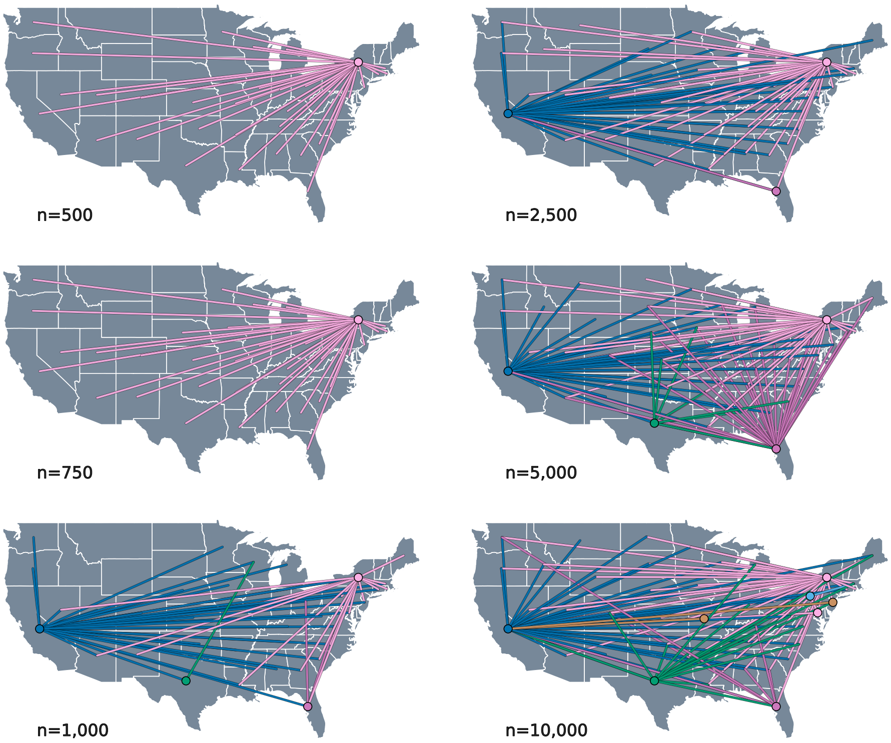

Figure S3. Connectivity plots for the PDA subsampled datasets. The geographical reconstructions from each MCC tree are projected into geographical space by drawing a line for each transition event, connecting origin (circle) and destination. The connectivity plot for the 250 taxa tree is not included in this plot because it was found to be similar to those of the 500, 750 and 1,000 taxa datasets.

142 4. Number of predictors included by subsample

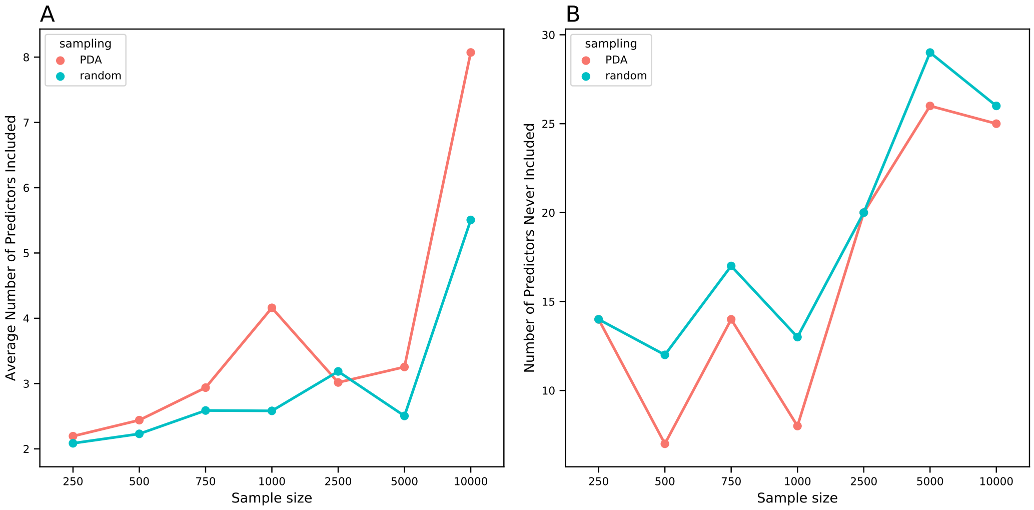

Figure S4. (**a**) Mean number of predictors included in the model by sample size and sampling scheme. As the number of sequences grows, the number of predictors included grows. (**b**) Number of predictors that never get included in the model. As the number of sequences grows, the number of predictors that never get included in the model also grows. This suggests reduced uncertainty with increasing data but we are unable to decouple this effect from the fact that the analysis is conditioned only on a single tree topology for the large sample sizes.

5. Evaluation of predictors on the randomly subsampled datasets

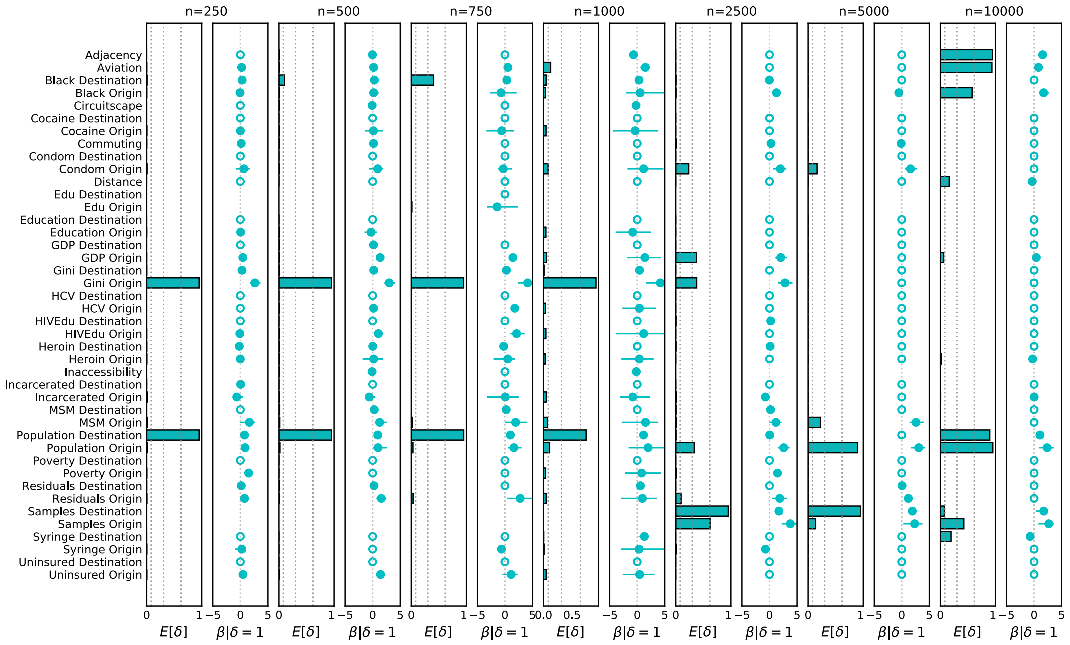

Figure S5. Predictors of HIV diffusion between US states for subsamples of 250, 500, 750, 1000, 2500, 5000 and 10000 taxa, selected using the random sampling scheme. **Bar plots** show the expecation of the GLM indicators associated to each explanatory variable, and represent the inclusion probability of each predictor. Indicator expectations corresponding to Bayes Factors of 5, 25, and 100 are represented with dotted vertical lines. **Point plots** show the mean and credible intervals of GLM coefficients on a log scale. The contribution of each predictor, when included in the model is given by $\beta|\delta = 1$. Predictors that were never included in the model are represented by dots with no filling.

6. Evaluation of predictors on the PDA subsampled datasets

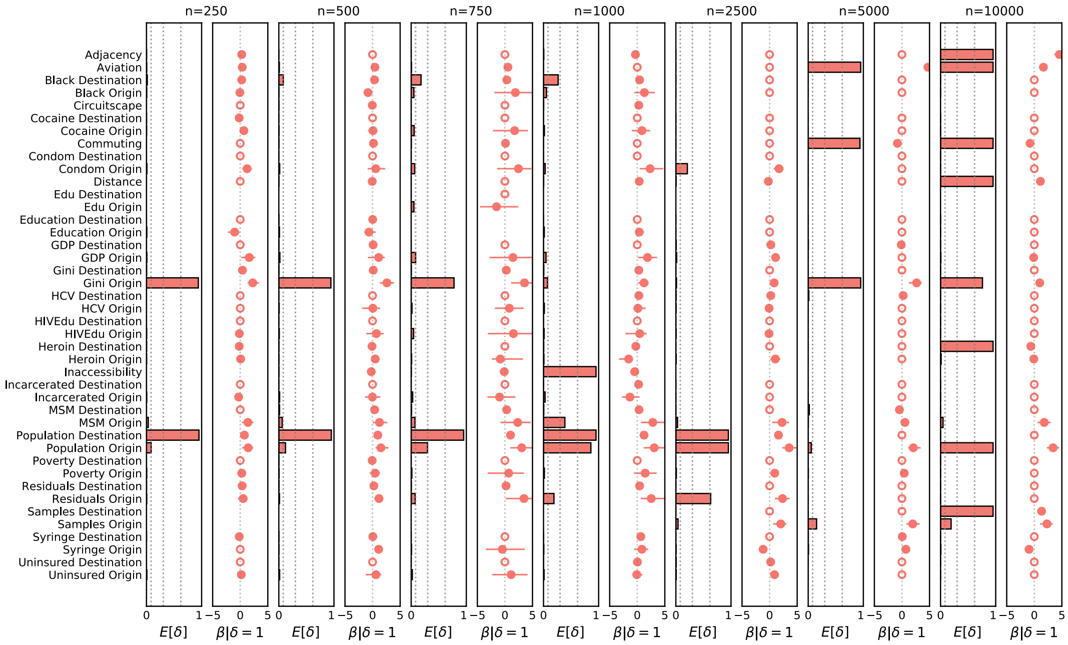

Figure S6. Predictors of HIV diffusion between US states for subsamples of 250, 500, 750, 1000, 2500, 5000 and 10000 taxa, selected using the Phylogenetic Diversity Analyzer sampling scheme. **Bar plots** show the expecation of the GLM indicators associated to each explanatory variable, and represent the inclusion probability of each predictor. Indicator expectations corresponding to Bayes Factors of 5, 25, and 100 are represented with dotted vertical lines. **Point plots** show the mean and credible intervals of GLM coefficients on a log scale. The contribution of each predictor, when included in the model is given by $\beta|\delta = 1$. Predictors that were never included in the model are represented by dots with no filling.

7. References

1. HIV Surveillance Reports Archive | Reports | Resource Library | HIV/AIDS | CDC. https://www.cdc.gov/hiv/library/reports/hiv-surveillance-archive.html, 2019. Accessed: 2019-7-21.
2. Bureau of Transportation Statistics. https://www.transtats.bts.gov/DL_SelectFields.asp?Table_ID=292. Accessed: 2019-8-5.
3. US Census Bureau. 2009-2013 5-Year American Community Survey Commuting Flows. https://www.census.gov/data/tables/time-series/demo/commuting/commuting-flows.html. Accessed: 2019-7-21.
4. County Distance Database. https://www.nber.org/data/county-distance-database.html. Accessed: 2019-7-21.
5. US Census Bureau. Geography Program. https://www.census.gov/programs-surveys/geography.html. Accessed: 2019-7-21.
6. Maps, Population Density Grid, v1: Global Rural-Urban Mapping Project v1 SEDAC. https://sedac.ciesin.columbia.edu/data/set/grump-v1-population-density/maps/services. Accessed: 2017-7-6.
7. Dellicour, S.; Vrancken, B.; Trovão, N.S.; Fargette, D.; Lemey, P. On the importance of negative controls in viral landscape phylogeography. *Virus Evol* **2018**, *4*, vey023.
8. McRae, B.H. Isolation by resistance. *Evolution* **2006**, *60*, 1551–1561.
9. McRae, B.H.; Dickson, B.G.; Keitt, T.H.; Shah, V.B. Using circuit theory to model connectivity in ecology, evolution, and conservation. *Ecology* **2008**, *89*, 2712–2724.

10. Weiss, D.J.; Nelson, A.; Gibson, H.S.; Temperley, W.; Peedell, S.; Lieber, A.; Hancher, M.; Poyart, E.; Belchior, S.; Fullman, N.; Mappin, B.; Dalrymple, U.; Rozier, J.; Lucas, T.C.D.; Howes, R.E.; Tusting, L.S.; Kang, S.Y.; Cameron, E.; Bisanzio, D.; Battle, K.E.; Bhatt, S.; Gething, P.W. A global map of travel time to cities to assess inequalities in accessibility in 2015. *Nature* **2018**, *553*, 333–336.
11. Dellicour, S.; Vrancken, B.; Trovão, N.S.; Fargette, D.; Lemey, P. On the importance of negative controls in viral landscape phylogeography. *Virus Evol* **2018**, *4*, vey023.
12. GDP and Personal Income. https://apps.bea.gov/itable/iTable.cfm?ReqID=70&step=1. Accessed: 2019-7-21.
13. US Census Bureau, Demographic Internet Staff. Current Population Survey (CPS), CPS Table Creator. https://www.census.gov/cps/data/cpstablecreator.html. Accessed: 2019-7-21.
14. Bureau, U.S.C. American factfinder-results. *Retrieved November* **2010**, *16*, 2016.
15. U.S. Statistics. https://www.hiv.gov/hiv-basics/overview/data-and-trends/statistics, 2019. Accessed: 2019-7-21.
16. Valera, P.; Chang, Y.; Lian, Z. HIV risk inside U.S. prisons: a systematic review of risk reduction interventions conducted in U.S. prisons. *AIDS Care* **2017**, *29*, 943–952.
17. Grey, J.A.; Bernstein, K.T.; Sullivan, P.S.; Purcell, D.W.; Chesson, H.W.; Gift, T.L.; Rosenberg, E.S. Estimating the Population Sizes of Men Who Have Sex With Men in US States and Counties Using Data From the American Community Survey. *JMIR Public Health Surveill* **2016**, *2*, e14.
18. Bureau of Justice Statistics (BJS) - Publications & Products: Prisoners. https://www.bjs.gov/index.cfm?ty=pbse&sid=40. Accessed: 2019-7-21.
19. Results | School Health Profiles | Data | Adolescent and School Health | CDC. https://www.cdc.gov/healthyyouth/data/profiles/results.htm, 2018. Accessed: 2019-7-21.
20. Bureau, U.S.C. American Fact Finder. https://factfinder.census.gov/faces/tableservices/jsf/pages/productview.xhtml?pid=ACS_10_1YR_B19083&prodType=table, 2010. Accessed: 2019-7-10.
21. Rosenberg, E.S.; Hall, E.W.; Sullivan, P.S.; Sanchez, T.H.; Workowski, K.A.; Ward, J.W.; Holtzman, D. Estimation of State-Level Prevalence of Hepatitis C Virus Infection, US States and District of Columbia, 2010. *Clin. Infect. Dis.* **2017**, *64*, 1573–1581.
22. amfAR :: Delivering Harm Reduction Services Including Syringe Exchange :: The Foundation for AIDS Research :: HIV / AIDS Research. https://www.amfar.org/endtheban/. Accessed: 2019-7-21.
23. Jalal, H.; Buchanich, J.M.; Roberts, M.S.; Balmert, L.C.; Zhang, K.; Burke, D.S. Changing dynamics of the drug overdose epidemic in the United States from 1979 through 2016. *Science* **2018**, *361*.
24. Multiple Cause of Death Data on CDC WONDER. https://wonder.cdc.gov/mcd.html. Accessed: 2019-7-21.
